# Novel diagnostic biomarkers of oxidative stress, immunological characterization and experimental validation in Alzheimer’s disease

**DOI:** 10.18632/aging.205084

**Published:** 2023-10-05

**Authors:** Di Hu, Xiaocong Mo, Luo Jihang, Cheng Huang, Hesong Xie, Ling Jin

**Affiliations:** 1 Department of Neurology and Stroke Centre, The First Affiliated Hospital of Jinan University, Guangzhou, China; 2 Department of Oncology, The First Affiliated Hospital of Jinan University, Jinan University, Guangzhou, China; 3 Department of Infectious Diseases, Affiliated Hospital of Zunyi Medical University, Zunyi, China; 4 Department of Traditional Chinese Medicine, The First Affiliated Hospital of Jinan University, Jinan University, Guangzhou, China

**Keywords:** Alzheimer’s disease, oxidative stress, molecular clusters, prediction model, immune infiltration

## Abstract

Alzheimer’s disease (AD) is a neurodegenerative condition causing cognitive decline. Oxidative stress (OS) is believed to contribute to neuronal death and dysfunction in AD. We conducted a study to identify differentially expressed OS-related genes (DEOSGs) through bioinformatics analysis and experimental validation, aiming to develop a diagnostic model for AD. We analyzed the GSE33000 dataset to identify OS regulator expression profiles and create molecular clusters (C1 and C2) associated with immune cell infiltration using 310 AD samples. Cluster analysis revealed significant heterogeneity in immune infiltration. The ‘WGCNA’ algorithm identified cluster-specific and disease-specific differentially expressed genes (DGEs). Four machine learning models (random forest (RF), support vector machine (SVM), generalized linear model (GLM) and extreme gradient boosting (XGB)) were compared, with GLM performing the best (AUC = 0.812). Five DEOSGs (NFKBIA, PLCE1, CLIC1, SLCO4A1, TRAF3IP2) were identified based on the GLM model. AD subtype prediction accuracy was validated using nomograms and calibration curves. External datasets (GSE122063 and GSE106241) confirmed the expression levels and clinical significance of important genes. Experimental validation through RT-qPCR showed increased expression of NFKBIA, CLIC1, SLCO4A1, TRAF3IP2, and decreased expression of PLCE1 in the temporal cortex of AD mice. This study provides insights for AD research and treatment, particularly focusing on the five model-related DEOSGs.

## INTRODUCTION

Alzheimer’s disease (AD) is a neurodegenerative disorder that typically manifests in middle-aged or elderly individuals. It is the most prevalent type of dementia among older adults, responsible for 60-80% of all cases, as reported by the Alzheimer’s Association of America [1]. AD is characterized by symptoms such as memory loss, language impairment, diminished thinking skills and behavioral changes, which eventually lead to severe cognitive impairment and loss of mobility [2, 3]. The cause of AD is still unknown and there is no complete cure available. Currently, medication and behavioral therapy can only help control the symptoms of the disease. Therefore, early diagnosis and intervention are crucial for patients. Amyloid β-protein (Aβ) is an abnormal protein that deposits in the brains of AD patients, leads to neuronal damage and cell death. Research has demonstrated that Aβ can produce reactive oxygen species (ROS), which are harmful chemicals that cause oxidative stress (OS), resulting in the death of nerve cells and impairing the structure and function of brain tissue [4]. Some studies have proved the important role of OS in AD. For example, the deficiency of Sirtuin-3 (SIRT3), a mitochondrial deacetylase, has been found to cause hyperactivity, reduced survival, and increased oxidative stress in cultured neurons. However, inhibiting ROS levels may reverse these effects. These findings highlight the importance of SIRT3 in regulating neuronal excitability in Alzheimer’s disease, particularly in relation to oxidative stress [5]. Studies have found that dioscin can reduce oxidative stress and inflammatory response in Alzheimer’s disease by down-regulating the expression levels of RAGE and NOX4, and up-regulating the expression levels of Nrf2 and HO-1. Additionally, dioscin inhibits p-NF-κB (p-p65)/NF-κB(p65), AP-1, and inflammatory factors involved in inflammatory pathways [6]. In a mouse model of Alzheimer’s disease induced by streptozotocin (STZ), activation of the G-protein-coupled receptor 55 (GPR55) has been found to have neuroprotective effects. Specifically, GPR55 activation was shown to reduce oxidative stress, neuroinflammation, and synaptic dysfunction in mice [7]. The above studies have demonstrated the significant role of OS in AD. However, there are few studies to further explore the impact of multiple OS-related genes on the diagnosis, treatment and prognosis of AD.

In this study, we identified molecular markers linked to OS and developed a diagnostic model with high sensitivity and specificity. This model can aid in the diagnosis and monitoring of AD. These findings enhance our understanding of the disease’s pathogenesis and offer a reference for the development of more accurate biomarkers and diagnostic methods. In future research, we will delve deeper into the biological functions and clinical applications of these molecular markers. This will enable us to provide improved support for the early detection and treatment of AD.

## RESULTS

### Dysregulation of OS regulators in AD patients

**Figure 1:**
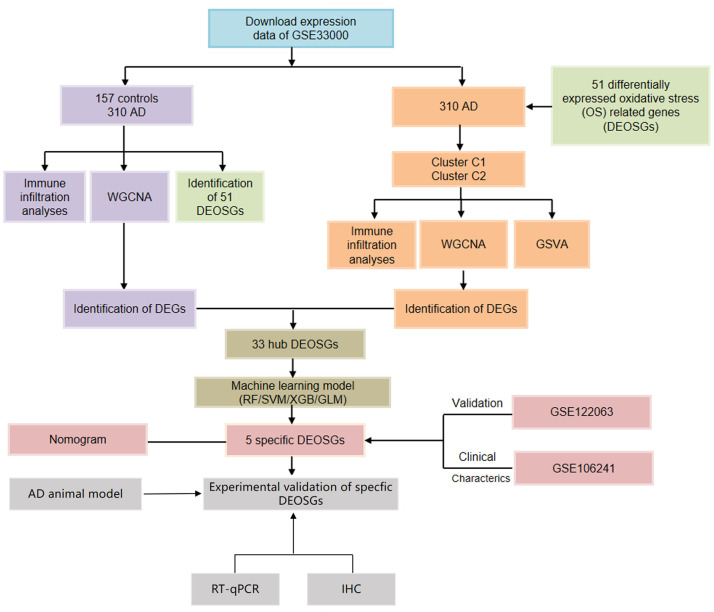
Flow chart of the study.

**Figure 2:**
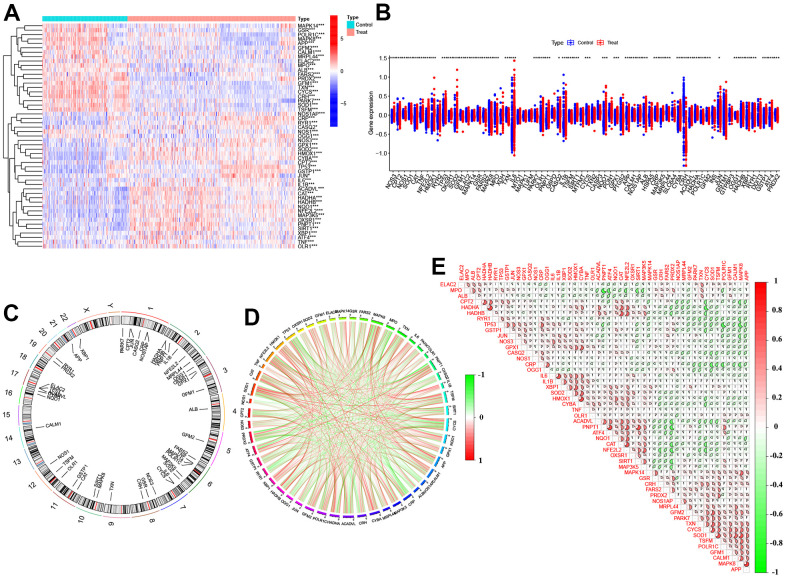
**Identification of dysregulated 51 DEOSGs in AD.** (**A**) Heatmap presented the 51 DEOSGs. (**B**) Boxplots showed the expression of 51 DEOSGs between Treat (AD) and Control (non-AD). (**C**) The location of 51 DEOSGs on chromosomes. (**D**) Gene relationship network diagram of 51DEOSGs. (**E**) Correlation analysis of 51 DEOSGs.

To investigate the role of OS regulators in the development of AD, we analyzed the expression profiles of 83 OS regulators in AD patients and non-AD controls from the GSE33000 dataset using ‘differ.R’ software. The entire study procedure is illustrated in Figure 1. Initially, we identified 51 differentially expressed OS regulators (DEOSGs). The study found that certain genes (NOS3, SOD2, IL6, OLR1, etc.) were expressed at higher levels in the cortex tissues of individuals with AD compared to those without the disease. On the other hand, the expression levels of other genes (MPO, MAPK8, GSR, POLR1C, etc.) were significantly lower in the cortex tissues of individuals with AD. The distribution of these differentially expressed genes on chromosomes is shown in Figure 2C. Subsequently, we conducted a correlation analysis to investigate whether OS regulators play a critical role in the development of AD. Our findings revealed synergistic effects between HADHB and HADHA, IL1β and IL6, CAT and NFE2L2, while MPO and PNPT1, ACADVL and PRDX2, TP53 and POLR1C showed antagonistic effects (Figures 2D, 2E). These results suggest a potential relationship among these DEOSGs.

### Identification of OS clusters in AD

**Figure 3:**
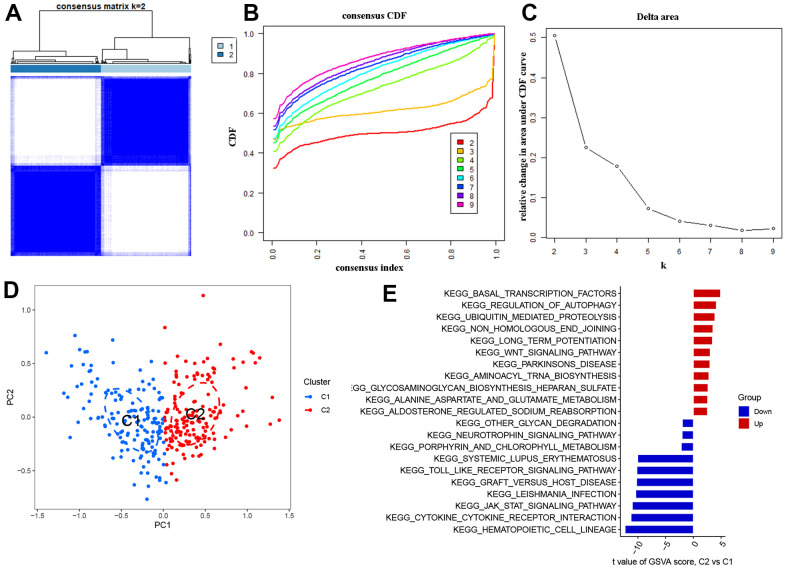
**Identification of OS molecular clusters in AD.** (**A**) Consensus clustering matrix when k = 2. (**B**, **C**) CDF delta area curves and the score of consensus clustering. (**D**) t-SNE analysis. (**E**) GSVA analysis between Cluster1 and Cluster2.

The AD samples were classified using the ‘cluster R’ algorithm based on the expression of the 51 DEOSGs mentioned earlier. When the k value was set to 2, the resulting cluster model was the most stable and effectively separated the samples into two clusters, C1 and C2 (as shown in Figures 3A). The CDF curve remained within a reasonable range of the consensus index (0.2-0.8) during this process (as depicted in Figures 3B, 3C). The output of tSNE analysis confirmed the previously observed difference between the two clusters (Figure 3D). To further investigate the functional characteristics of the clusters, we conducted GSVA analysis, which revealed that Cluster 2 was enriched in ‘basal transcription factors’, ‘regulation of autophagy’, and ‘ubiquitin mediated proteolysis’, while Cluster 1 showed upregulation of ‘hematopoietic cell lineage’, ‘cytokine receptor’, and ‘JAK-STAT signaling pathway’ (Figure 3E).

### Analysis of immune infiltration characteristics in AD patients and OS clusters

**Figure 4:**
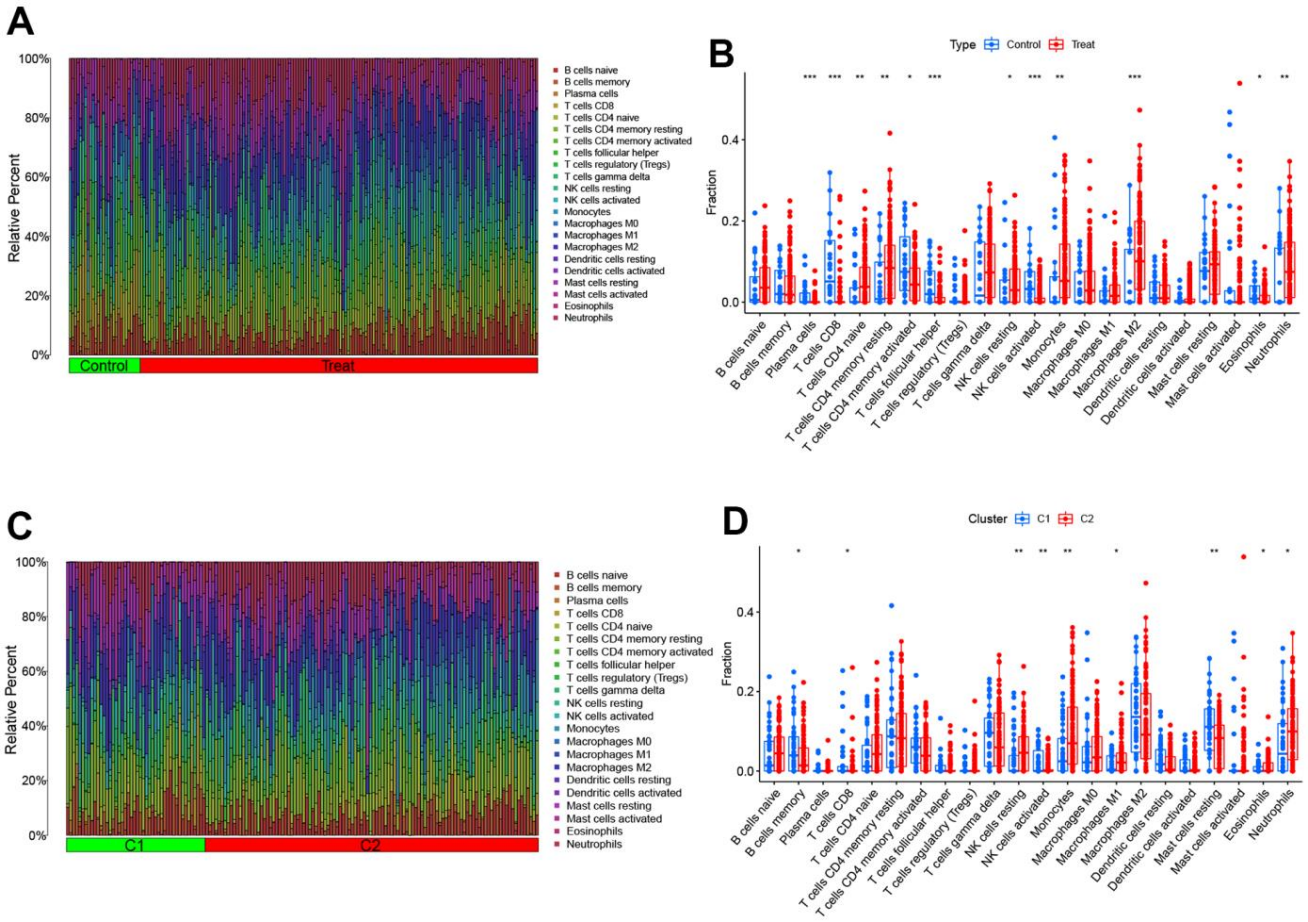
**Comparison of OS molecular clusters and AD patients in terms of immune characteristics.** (**A**, **B**) The relative abundances of 22 infiltrated immune cells between AD samples and controls. (**C**, **D**) The relative abundances of 22 infiltrated immune cells between two OS molecular clusters. *p < 0.05; **p < 0.01; ***p < 0.001.

To investigate the variation of AD in the immune system, we utilized the CIBERSORT algorithm to compare the proportions of 22 different immune cell types in two groups - AD and control (Figure 4A). The results showed that AD patients had a higher infiltration degree of T cells CD4 naive, T cells CD4 memory resting, NK cells resting, Monocytes, Macrophages M2, and Neutrophils (Figure 4B). These findings suggest that immunomodulation may play a crucial role in the occurrence of AD. Meanwhile, the study revealed distinct immune characteristics between OS Cluster1 and Cluster2, as shown in Figure 4C. Cluster 2 had relatively higher levels of NK cells resting, Monocytes, Macrophages M1, Eosinophils and Neutrophils, while Cluster 1 had higher abundance of B cells memory, T cells CD8, NK cells activated and Mast cells resting, as illustrated in Figure 4D.

### Development of WGCNA co-expression network and module detection

**Figure 5:**
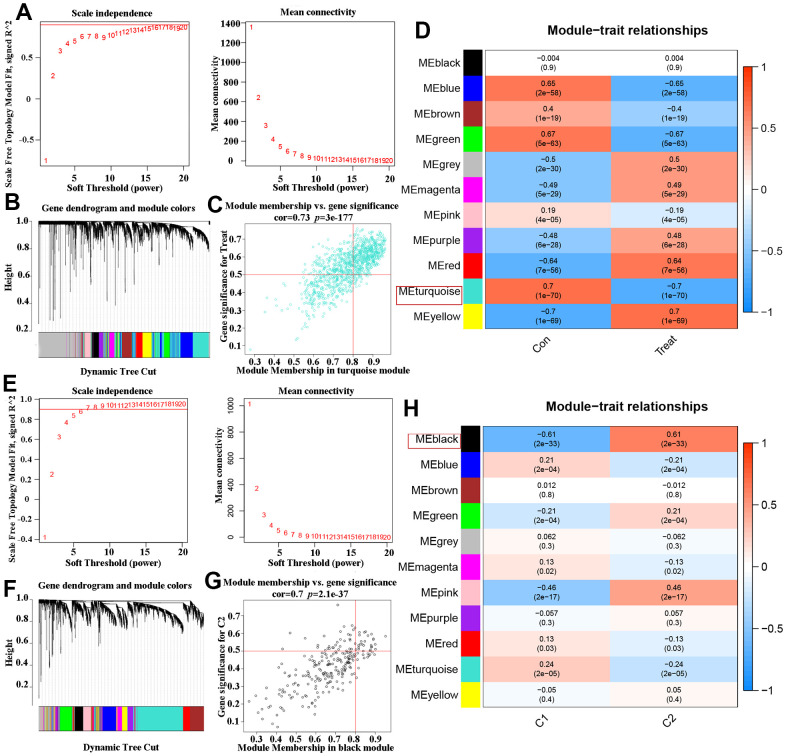
**Co-expression network of DEGs in AD and two DEOSGs clusters.** (**A**) The detection of soft threshold power in AD. (**B**) Cluster tree dendrogram of co-expression modules in AD patients. (**C**) Scatter plot in ME turquoise module and the DEGs significance for AD. (**D**) Heatmap of association of module feature genes with several clinical states. (**E**) The detection of soft threshold power in two DEOSGs clusters. (**F**) Cluster tree dendrogram of co-expression modules in the two DEOSGs clusters. (**G**) Scatter plot ME black module and the DEGs significance for the two DEOSGs clusters. (**H**) Correlation heatmap between two DEOSGs clusters.

In order to analyze the major gene modules of AD, the co-expression network and prospective modules were established using the “WGCNA” algorithm. The top 25% genes with the highest variance (2439 genes) were clustered and merged into eleven distinct co-expression modules (Figure 5A, 5B). The correlation between the 11 color modules and typical clinical features (i.e., AD and ND) was analyzed using Pearson’s correlation analysis. Interestingly, the ME turquoise module, consisting of 759 genes, showed a strong correlation with the ‘Treat (AD)’ trait. Additionally, a positive relationship was observed between the two (Figure 5C, 5D). The ‘WGCNA’ algorithm was continuously used to identify and construct important gene modules related to OS clusters. The exploration process was consistent with previous analyses, and 11 different models were identified as significant co-expression modules (Figure 5E, 5F). In the clinical features module, there was a strong correlation between the ME black turquoise module (which contains 245 genes) and OS Cluster1 and OS Cluster2. This was demonstrated in Figure 5G, 5H. Based on this finding, certain key genes from the ME turquoise module that are associated with Alzheimer’s disease and the ME black turquoise module that are related to OS Clusters were selected for further analysis.

### Identification of the specific DEGs through machine learning models

**Figure 6:**
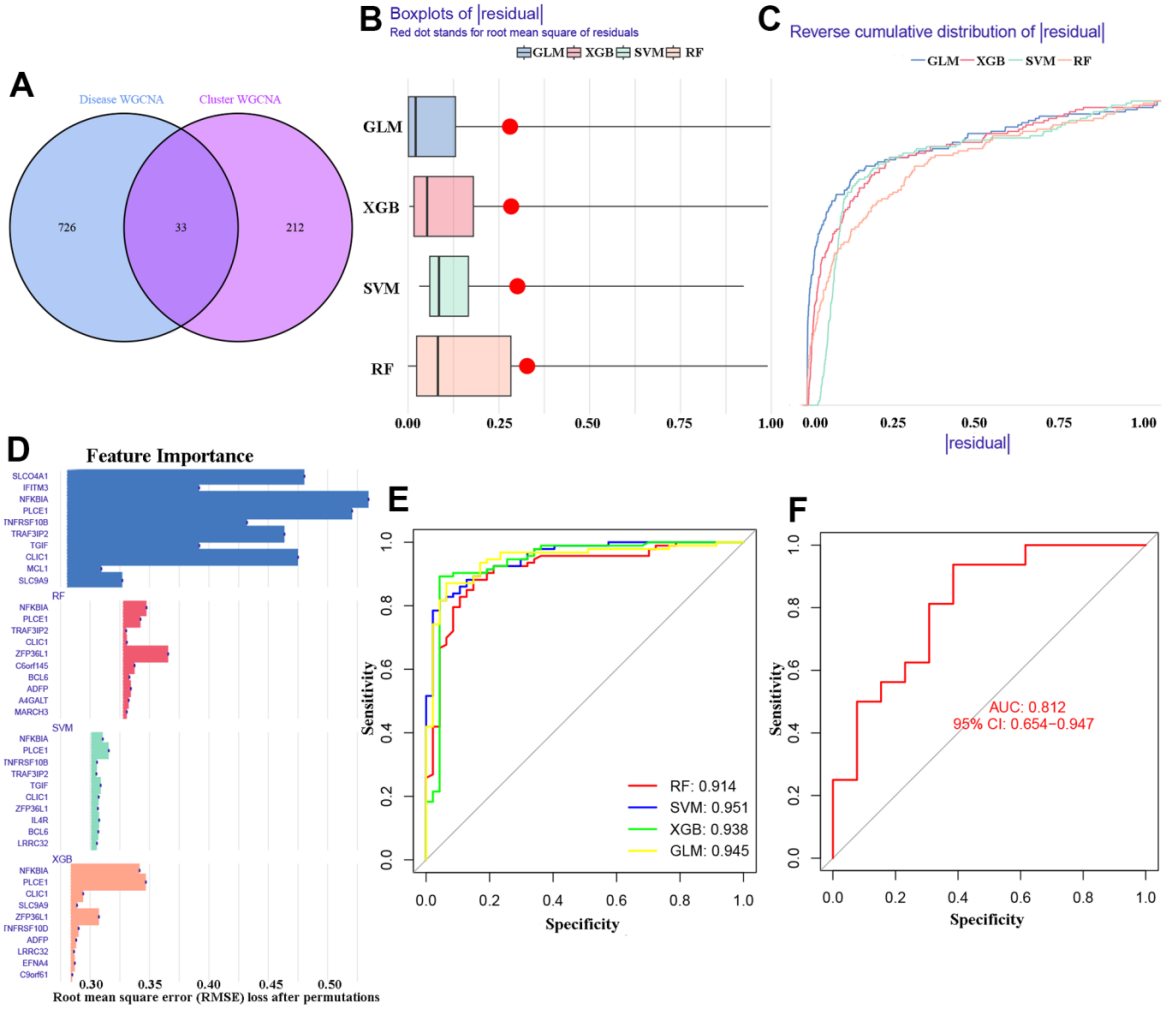
**Identification of cluster-specific DEGs of machine models.** (**A**) 33 overlapping DEGs of two WGCNA co-expression. (**B**, **C**) Residuals for the four machine learning models. (**D**) The important features in the four machine models. (**E**) ROC analysis. (**F**) The AUC values of GLM model in GSE122063 dataset.

The study identified 33 overlapping differentially expressed genes (DEGs) from two WGCNA co-expression results (Figure 6A). These 33 DEGs were used to establish four machine learning models (RF, SVM, GLM, and XGB) using the ‘model R’ package. The results in Figure 6B, 6C show that the GLM and XGB models had relatively lower residual regulation. Subsequently, the top 10 important feature DEGs of each online machine learning models were ranked and presented in Figure 6D using root mean square error (RMSE) analysis. Additionally, we evaluated the efficacy and sensitivity of the four learning algorithms by calculating the receiver operating characteristic (ROC) curve based on 5-fold cross-validation. The study found that SVM and GLM algorithms had a high area under the ROC curve (AUC) with SVM having an AUC of 0.951, GLM having an AUC of 0.945, XGB having an AUC of 0.938, and RF having an AUC of 0.914 (Figure 6E). Based on these results, the GLM model was identified as the best method for distinguishing AD patients with OS clusters. Additionally, the top five important DEGs (NFKBIA, PLCE1, CLIC1, SLCO4A1, and TRAF3IP2) from the model were selected for further analysis. Finally, the GLM model for GSE122063 dataset showed an AUC value of 0.812 with a 95% confidence interval of 0.654 - 0.947. This indicates that our diagnosis model has a high ability to distinguish between individuals with AD and normal individuals, as displayed in Figure 6F.

### Construction of the nomogram model and immune cell infiltration

**Figure 7:**
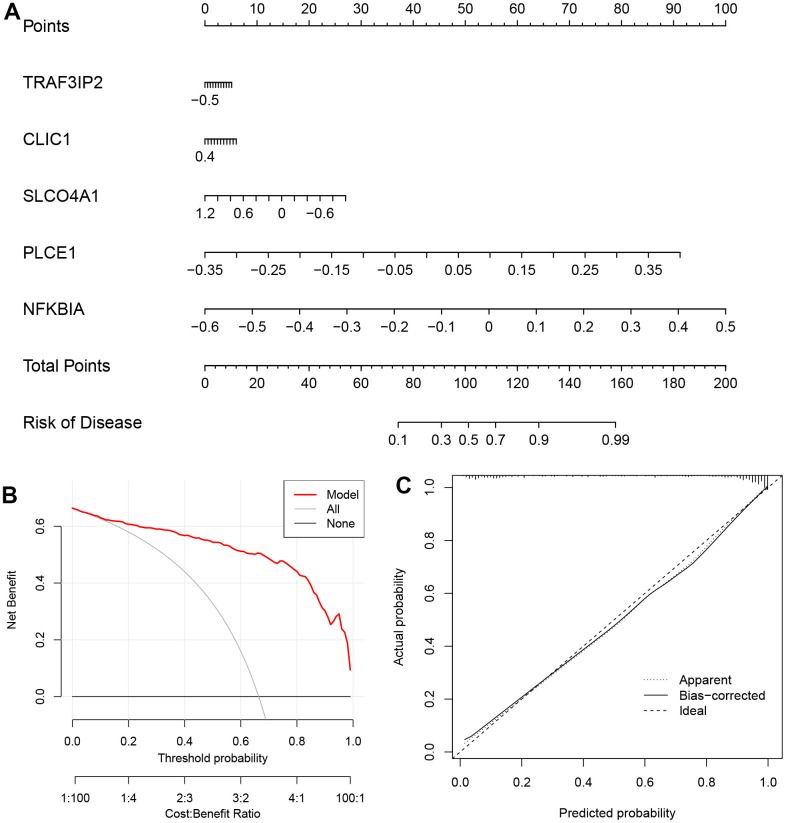
**The nomogram model (based on the five DEOSGs) in GSE122063.** (**A**) The nomogram. (**B**) The DCA curve. (**C**) The curve of calibration.

**Figure 8:**
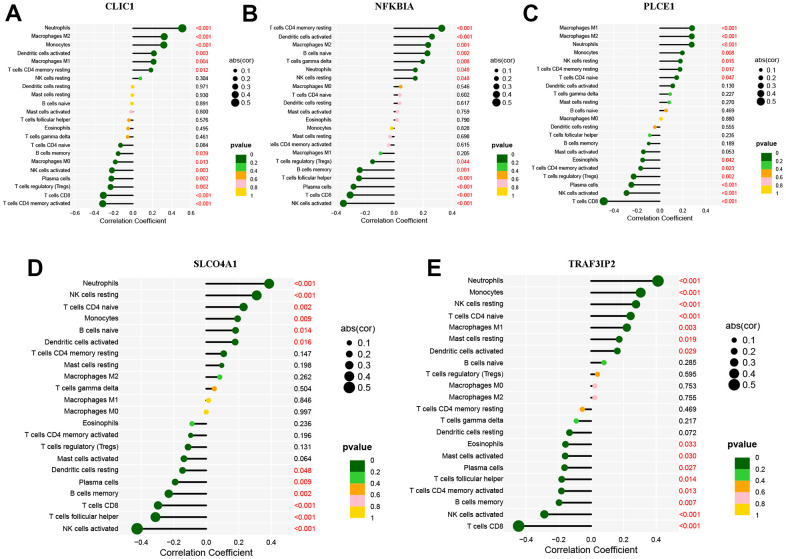
**Immune cell infiltration of the five DEOSGs.** (**A**) The correlation analysis of immune cell infiltration with CLIC1. (**B**) The correlation analysis of immune cell infiltration with NFKBIA. (**C**) The correlation analysis of immune cell infiltration with PLCE1. (**D**) The correlation analysis of immune cell infiltration with SLCO4A1. (**E**) The correlation analysis of immune cell infiltration with TRAF3IP2.

The ‘rms’ package was used to construct a nomogram model based on five specific genes (NFKBIA, PLCE1, CLIC1, SLCO4A1 and TRAF3IP2) to evaluate and predict the risk of AD. The calibration curve showed high predictive accuracy for the nomogram model, as depicted in Figure 7B, 7C. We further investigated the correlation between these five DEOSGs and inflammation, as shown in Figure 8A–8E, which confirmed the correlation between five diagnostic DEOSGs and 22 immune cells. The study found a positive correlation between CLIC1 and immune cells, specifically Neutrophils and Macrophages M2. However, there was a negative correlation between CLIC1 and T cells CD4 memory activated (Figure 8A). Additionally, the results showed that NFKBIA was positively correlated with T cells CD4 memory resting, but negatively correlated with NK cells activated (Figure 8B). Finally, PLCE1 was found to be positively correlated with Macrophages M1 and M2, but negatively correlated with T cells CD8 (Figure 8C). In Figure 8D, SLCO4A1 showed a positive correlation with Neutrophils and NK cells resting, but a negative correlation with NK cells activated. Similarly, in Figure 8E, TRAF3IP2 exhibited a positive correlation with Neutrophils and Monocytes, but a negative correlation with NK cells activated and T cells CD8.

### Relationships of hub DEOSGs and clinical characteristics

**Figure 9:**
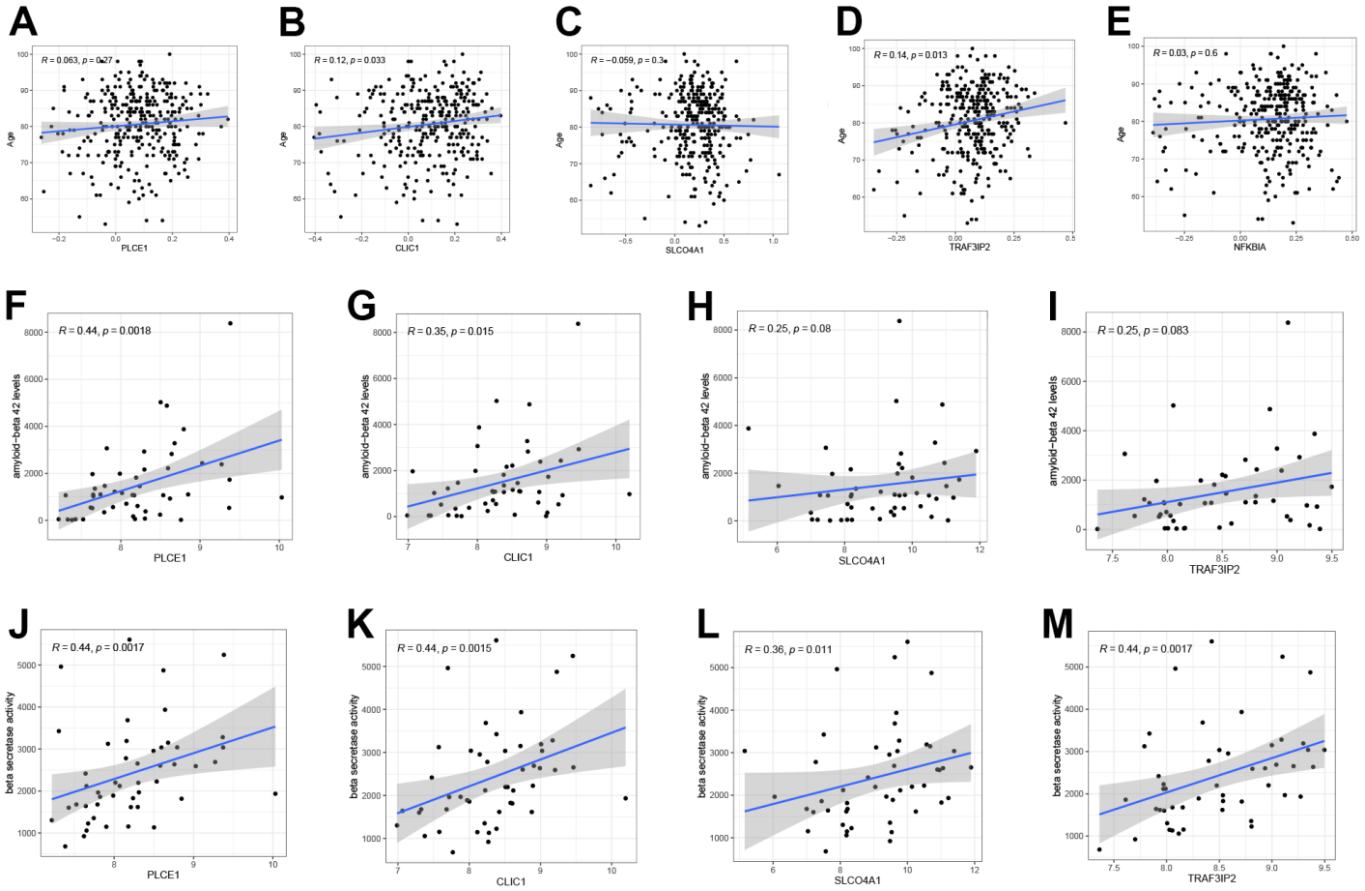
**The correlation between five DEOSGs and clinical characteristics.** (**A**–**E**) Correlation between PLCE1 (**A**), CLIC1 (**B**), SLCO4A1 (**C**), TRAF3IP2 (**D**), NFKBIA (**E**) and age (GSE33000). (**F**–**I**) Correlation between PLCE1 (**F**), CLIC1 (**G**), SLCO4A1 (**H**), TRAF3IP2 (**I**) and Aβ-42 (GSE106241). (**J**–**M**) Correlation between PLCE1 (**J**), CLIC1 (**K**), SLCO4A1 (**L**), TRAF3IP2 (**M**) and beta-secretase activity (GSE106241).

The relationships between five DEOSGs and clinical characteristics age (GSE33000), beta-secretase activity and Aβ-42 levels (GSE106241, NFKBIA was not discovered in the dataset) were depicted in Figure 9. Three DEOSGs (PLCE1, SLCO4A1, and NFKBIA) did not show any significant difference, whereas CLIC1 and TRAF3IP2 exhibited a positive correlation with age (CLIC1, R = 0.12; TRAF3IP2, R = 0.14) as depicted in Figure 9A–9E. The study found that, apart from SLCO4A1 and TRAF3IP2, the other DEOSGs (PLCE1, R = 0.44; CLIC1, R = 0.35) showed a positive correlation with Aβ-42 level (Figure 9F–9I). Additionally, all four DEOSGs were found to be positively associated with beta-secretase activity (Figure 9J–9M).

### Validation of DEOSGs by other GEO datasets and experimental validation in AD animal samples

**Figure 10:**
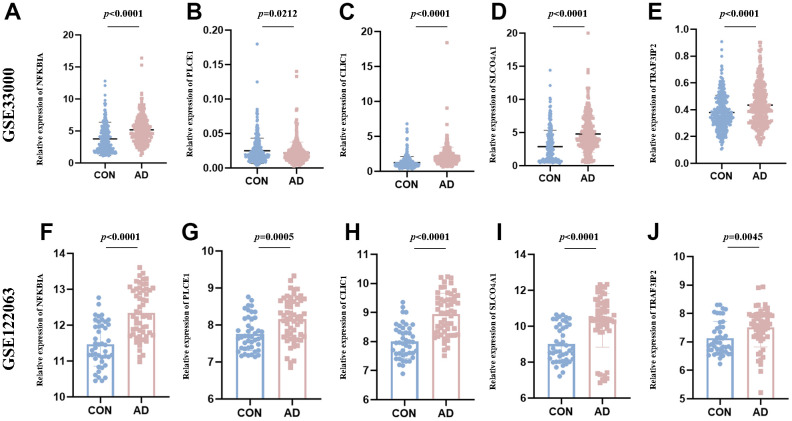
**The expression value of NFKBIA, PLCE1, CLIC1, SLCO4A1 and TRAF3IP2.** (**A**–**E**) Validated in the GSE33000. (**F**–**J**) Validated in the GSE122063. *p < 0.05; **p < 0.01; ***p < 0.001.

**Figure 11:**
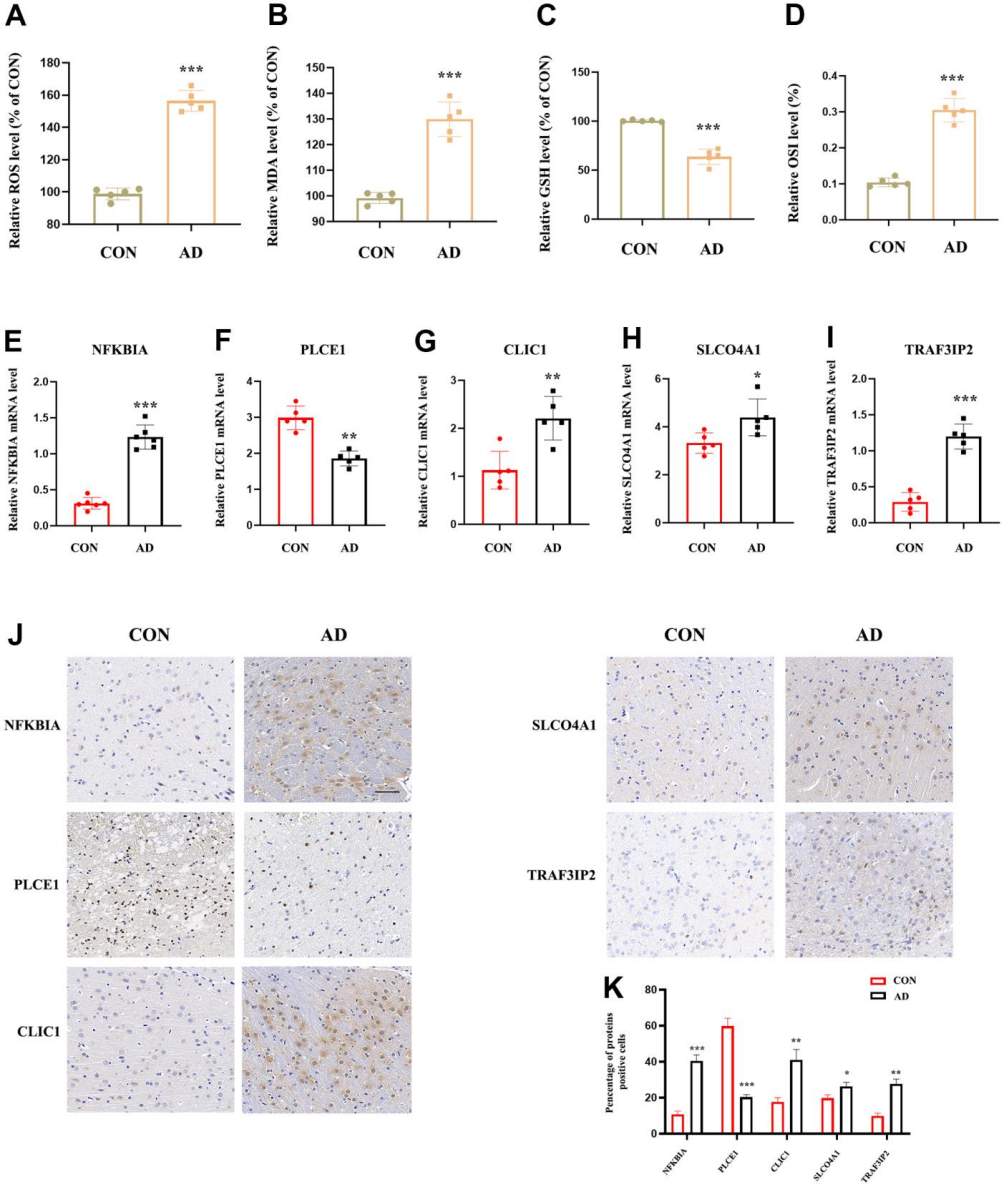
**The expression value of NFKBIA, PLCE1, CLIC1, SLCO4A1 and TRAF3IP2 in AD animal model.** (**A**) Detection of ROS level in the cortex of mice brain. (**B**) Detection of MDA level in the cortex of mice brain (*n* = 5). (**C**) Detection of GSH level in the cortex of mice brain (*n* = 5). (**D**) Detection of OSI level in the cortex of mice brain (*n* = 5). (**E**–**I**) RT-qPCR for NFKBIA, PLCE1, CLIC1, SLCO4A1 and TRAF3IP2 in the cortex of mice brain (*n* = 5). (**J**, **K**) IHC for NFKBIA, PLCE1, CLIC1, SLCO4A1 and TRAF3IP2 in the mice brain. *p < 0.05; **p < 0.01; ***p < 0.001. OSI: Oxidative stress index.

The study assessed the expression value of model-related specific DEOSGs (NFKBIA, PLCE1, CLIC1, SLCO4A1, and TRAF3IP2) in the GSE33000 and GSE122063 datasets, which served as the validation set for AD. The findings indicated that, with the exception of PLCE1, which did not show the same trend (down-regulated in GSE33000 and up-regulated in GSE122063), the other four DEOSGs were elevated in both datasets (Figure 10A–10J). Based on this, we further used APP/PS1 mice (AD group) and C57BL/6 mice (control) for *in vitro* experimental verification. Firstly, we detected the levels of MDA, ROS, GSH and OSI in the mice cortex. The results showed a significant increase in ROS (Figure 11A), MDA (Figure 11B) and OSI (Figure 11D) levels, while an obvious decrease in GSH level of AD mice (Figure 11C), indicating that the pathogenesis of AD is closely related to the activation of oxidative stress. Next, to deeply verify the reliability of the five DEOSGs in the mice cortex of AD, RT-qPCR was conducted. The results showed a significant enhancement in the expression of NFKBIA, CLIC1, SLCO4A1 and TRAF3IP2, while the expression of PLCE1 was observed to be decreased in the AD model (Figure 11E–11I). Additionally, to quantitatively assess the internal index in AD model, brain sections were stained for the expression of these five proteins. To evaluate the internal index in AD model, brain sections were stained for the expression of five proteins. The results showed that the AD group had higher staining for NFKBIA, CLIC1, SLCO4A1, and TRAF3IP2, while PLCE1 had lower staining compared to the control (Figure 11J, 11K). Based on these findings, we conclude that these specific DEOSGs have the potential to be effective diagnostic biomarkers for AD, supporting our previous speculations.

## DISCUSSION

Alzheimer’s disease is a degenerative disease that progresses over time, primarily affecting memory and cognitive function. This disease places a significant burden on the patient and their family. Currently, there are several challenges in treating AD, including the difficulty of early diagnosis, limited treatment options, and slow progress in research [8, 9]. To address the aforementioned challenges, it is imperative to reinforce fundamental research, explore novel diagnostic and therapeutic approaches and medications, and offer patients with more tailored medical services. For instance, by considering the patient’s unique characteristics and health status, healthcare providers can choose the most suitable treatment and medication to enhance the efficacy of the treatment while minimizing the likelihood of adverse reactions [10, 11]. Therefore, this study aims to identify more suitable oxidative stress molecular clusters for the early prediction and individualized treatment of Alzheimer’s Disease.

To achieve this, the study performed a comprehensive DEOSGs expression profiling by comparing the expression profiles of normal and AD patient brain tissues. The results showed that several DEOSGs were significantly different in expression between the two groups, indicating their potential for further investigation. To investigate the potential interaction between DEOSGs in AD, we performed a correlation analysis and identified some DEOSGs that exhibited synergistic or antagonistic effects. We then used unsupervised cluster analysis to group the 51 DEOSGs into two clusters (C1 and C2) based on their expression levels. To further understand the functional differences between these clusters, we conducted GSVA analysis and found that cluster C2 was enriched with “basal transcription factors”, “autophagy regulation” and “ubiquitin-mediated proteolysis”. In contrast, “cytokine receptors” and “JAK-STAT signaling pathway” were upregulated in C1. These above pathways are related to the formation and development of AD [12, 13]. These aforementioned pathways are associated with the formation and progression of AD. The presence of distinct enriched pathways may lead to varying prognoses for patients with AD. By determining the cluster to which a patient belongs, we can make a preliminary assessment of their potential prognosis. At the same time, AD patients have higher levels of T cell CD4 naive, T cell CD4 memory resting, NK cell resting, neutrophils and monocytes compared to healthy individuals. These findings are consistent with previous research and suggest a strong link between AD and the immune system [14]. In patients with Alzheimer’s disease, a significant increase in CD4+ T lymphocytes has been observed, indicating an alteration in the post-thymic maturation of antigen-specific lymphocytes. Specifically, the accumulation of effector memory and terminally differentiated lymphocytes is observed in AD patients compared to healthy controls. effector memory cells differentiate from central memory cells in the presence of antigens and cytokines such as IL-7. In the presence of a high antigenic load, effector memory cells further differentiate into terminally differentiated, which represent the end-stage effector cells. Therefore, the neuroinflammation associated with AD can be characterized as a complex impairment of the immune response, exhibiting a pronounced skewing towards inflammatory and effector responses [15, 16]. Evidence suggests that depletion of NK cells in triple transgenic AD mouse models can alleviate neuroinflammation, promote neurogenesis, and improve cognitive function. NK cell depletion does not significantly affect the concentration of beta-amyloid proteins but enhances neurogenesis and reduces neuroinflammation [17]. Zenaro et al. identified that in two transgenic models of Alzheimer’s disease (5xFAD and 3xTg-AD mice), neutrophil infiltration and localization within amyloid-beta (Aβ) deposits were accompanied by the release of neutrophil extracellular traps (NETs) and IL-17. Furthermore, the study highlighted the significance of LFA-1 integrin in regulating neutrophil extravasation into the central nervous system and their movement within the parenchyma. Depleting neutrophils or inhibiting their trafficking via LFA-1 blockade resulted in a reduction of Alzheimer’s disease-like neuropathology and an improvement in memory in cognitively impaired mice [18]. Yan et al. observed a higher proportion of peripheral monocytes contributing to the presence of macrophages in the choroid plexus, meninges, and perivascular spaces surrounding blood vessels in aged AD mice compared to normal control mice. This finding suggests an enrichment of potential sites for peripheral monocyte infiltration into the brain parenchyma. Notably, splenectomy significantly reduced circulating monocytes and diminished the abundance of plaque-associated macrophages derived from definitive hematopoiesis, resulting in an increased amyloid plaque load. These findings indicate that peripheral-derived monocytes infiltrate the brain parenchyma, targeting amyloid plaques to reduce plaque burden [19]. These results also provide support for the potential use of immunotherapy as a treatment for AD. Cluster 1 showed a higher abundance of B cell memory, T cell CD8, NK cell activation, and mast cell quiescence. Additionally, DEOSGs of cluster 1 are enriched in the JAK/STAT pathway, which plays a crucial role in promoting neuroinflammation in neurodegenerative diseases like AD [20]. This pathway initiates innate immunity, coordinates adaptive immune mechanisms, and ultimately suppresses neuroinflammatory responses. This suggests that patients with C1 may have a better prognosis.

To investigate the association between oxidative stress and AD pathogenesis, the levels of oxidative stress markers, including ROS, MDA, GSH, and OSI, were measured in the cortex of AD and control mice. The significant increase in ROS, MDA, and OSI levels, along with the noticeable decrease in GSH level, strongly suggests the activation of oxidative stress pathways in AD. Other studies have also observed the importance of biomarkers or indicators such as ROS, MDA, OSI, and GSH in the pathogenesis of AD. Under normal physiological conditions, reactive oxygen species (ROS) play crucial roles in cellular signal transduction and gene transcription activation. However, when the accumulation of ROS surpasses the antioxidant capacity, they can attack vital biomolecules within the cell, such as phospholipids, proteins, and nucleic acids, thereby disrupting normal cellular functions. This excessive ROS accumulation can stimulate mitochondria to produce pro-apoptotic proteins, leading to apoptotic cell death in neuronal cells within the central nervous system [21]. MDA, OSI, and GSH, among other biomarkers or indicators, are widely employed for evaluating the extent of oxidative damage to biomolecules. Sterubin, a flavonoid compound, has been identified as a neuroprotective agent capable of reducing the expression of inflammatory biomarkers (such as IL-6, IL-β, and TNF-α) as well as oxidative stress markers (such as SOD and MDA) in order to prevent chemically-induced Alzheimer’s disease in rats [22]. Within the brain system, GSH plays a pivotal role as an essential enzymatic antioxidant, serving as a defense mechanism against oxidative damage caused by reactive oxygen species (ROS) [23]. A prospective study, HELIAD, reported that higher baseline plasma GSH levels were associated with a reduced risk of developing Alzheimer’s disease (AD) and better preservation of longitudinal executive function [24].

When building a predictive model, machine learning algorithms utilize historical data to identify features associated with predictor variables [25]. In this study, we compared the prediction performance of machine learning classifiers, including RF, SVM, GLM, and XGB, based on the expression profiles of 33 hub DEOSGs obtained by intersection. Ultimately, we established a prediction model based on GLM. We constructed a 5-gene-based GLM model (NFKBIA, PLCE1, CLIC1, SLCO4A1 and TRAF3IP2) by selecting five important variables. One of these variables is NFκB inhibitor alpha (NFκBIα), which encodes a protein that interacts with REL dimers and inhibits the NFκB/REL complex involved in inflammatory responses. Studies have reported that loss-of-function mutations upstream of NF-κB affect NF-κB activity in AD patients, leading to anatomical defects such as shrinkage of the entorhinal cortex and early AD limbic system [26]. Phospholipase C epsilon 1 (PLCE1) is an enzyme that plays a crucial role in the regulation of various cellular processes such as cell growth, differentiation, and gene expression [27]. It catalyzes the hydrolysis of phosphatidylinositol-4,5-bisphosphate, which leads to the production of two second messengers that are responsible for regulating these processes. Recent studies have shown that in Alzheimer’s disease, the activation of chloride intracellular channel 1 (CLIC1) is necessary for β-amyloid-induced production of reactive oxygen species by microglia [28]. Additionally, Solute carrier organic anion transporter family member 4A1 plays a crucial role in the transportation of various compounds, such as sugars, bile salts, organic acids, metal ions, amines, and estrogens [29]. Another protein, TRAF3 interacting protein 2 (TRAF3IP2), regulates the response of members of the Rel/NF-κB transcription factor family to cytokines [30]. The pathway is crucial for AD. Our diagnostic model showed an AUC value of 0.812 and a 95% CI of 0.654-0.947 in the GSE122063 validation dataset, indicating high diagnostic efficacy and universal applicability. Therefore, we proceeded to use these five genes to develop a nomogram model for early diagnosis of AD, providing convenience to clinicians for timely intervention. The results of the immune cell correlation analysis in the external data set GSE106241 confirmed the relevance of these genes to the immune system, indicating the need for further investigation. Numerous studies have demonstrated that elevated levels of Aβ-42 and increased β-secretase activity are major pathological mechanisms contributing to the progression and poor prognosis of AD [31, 32]. Therefore, we performed a correlation analysis of genes from AD samples from GSE106241 with Aβ-42 levels and β-secretase activity. Our findings revealed that all predictive genes were positively correlated with Aβ-42 level (with the exception of TRAF3IP2 and SLCO4A1) and β-secretase activity with statistical significance. To confirm our findings, we analyzed the expression of five genes in two different datasets (GSE33000 and GSE122063). We observed that four of these DEOSGs were upregulated in both datasets, while only PLCE1 showed a different trend (downregulated in GSE33000 and upregulated in GSE122063). To further validate our bioinformatics predictions, we generated AD animal models and performed RT-qPCR on treated brain tissue samples. The results of the vivo experiment showed a significant increase in the levels of NFKBIA, CLIC1, SLCO4A1, and TRAF3IP2 mRNA, while the expression of PLCE1 was significantly decreased. These results are consistent with previous analyses. Therefore, the GLM model consisting of these five DEOSGs could be a reliable indicator for evaluating AD subtypes and pathological outcomes in AD patients.

## CONCLUSIONS

In conclusion, our study has revealed the correlation between OS-related genes and immune cell infiltration in AD patients with distinct OS clusters, highlighting the heterogeneity of the immune system. We have identified and validated five DEOSGs (NFKBIA, PLCE1, CLIC1, SLCO4A1, and TRAF3IP2) that can accurately predict the pathological outcome of AD patients. Our findings may provide new insights into the treatment and monitoring of AD. However, further studies are needed to uncover the underlying molecular mechanisms.

## MATERIALS AND METHODS

### Data acquisition

Gene expression data were obtained from the GEO database (http://www.ncbi.nlm.nih.gov/geo). The training dataset consisted of 157 controls and 310 AD cases, downloaded from the GSE33000 dataset (GPL4372 platform). Validation dataset was obtained from GSE122063 (GPL16699 platform). Clinical data from GSE106241 (GPL24170 platform) were used to validate the correlation of the genes in the final model.

### Analysis of differential expression genes

We utilized the “limma” R package to analyze the Differential Expression Genes (DEGs) between AD and control groups in GSE33000. The DEGs were selected based on the criteria of |log_2_FC| > 0.58 and *P*<0.05. Subsequently, we identified the differentially expressed OS-related genes (DEOSGs) from the DEGs list, utilizing the OS-related websites (https://www.genecards.org/) as a reference.

### Unsupervised clustering processing

In this study, we utilized unsupervised cluster analysis with the ‘ConsensusClusterPlus’ R package to classify AD patients based on previously obtained sets of DEOSGs. The k-means algorithm was used with 1,000 iterations to assign 310 AD samples into different clusters. We conducted a study where we chose the maximum number of subtypes (k=9) and evaluated the optimal number of clusters using cumulative distribution function (CDF) curves, consensus matrix, and consensus clustering score (>0.9). To analyze the data, we performed a principal component analysis (PCA) with the “factoextra” R package. Prior to this, we standardized and normalized GSE33000 using the function normalize between arrays in the “limma” R package. To illustrate significantly deregulated genes, we utilized the ‘ggplot2’ and ‘ComplexHeatmap’ packages to generate volcano plots and heat maps, respectively.

### Gene set variation analysis (GSVA) analysis

The GSVA is a technique utilized to analyze gene expression profiling data. It aids in the identification of unique gene expression patterns and assesses the activity levels of specific biological processes or pathways in each sample. The ‘c2.cp.kegg.v7.4.symbols’ file was obtained from the MSigDB database (https://www.gsea-msigdb.org/gsea/msigdb/) and used in conjunction with the ‘GSVA’ package in R to perform GSVA enrichment analysis. This allowed for the identification of enriched gene sets among different DEOSGs clusters, which were then displayed as histograms.

### Gene expression profiles of immune cell infiltration in AD

To determine the relative abundance of infiltrating immune cells in various populations, we utilized the “CIBERSORT” algorithm. This algorithm estimated the potential proportion of infiltrating immune cells by referencing a set of 22 sorted immune cell subtypes for each sample in the control and treatment groups, as well as the C1 and C2 cohorts. In CIBERSORT, deconvolution P-values are calculated for each sample using a Monte Carlo sampling method to estimate reliability. We performed 1000 permutations and generated corresponding P values.

### Validation of co-expression genes

In this study, the ‘WGCNA’ R package was utilized to cluster genes based on their expression patterns in GSE33000, leading to the formation of distinct modules. The focus was on analyzing the relationship between genes and phenotypes/traits within these modules. To construct a co-expression network, the top 25% of genes in the dataset were analyzed for variance. A weighted adjacency matrix is first generated, followed by its conversion into a topological overlap matrix (TOM). Using the TOM difference metric (1-TOM), modules are obtained through a hierarchical clustering tree algorithm with a minimum module size of 100. Meanwhile, we utilized two types of significance measures: module significance (MS) and gene significance (GS). MS describes the relationship between modules and disease states, while GS describes the correlation between genes and clinical phenotypes. To identify possible correlations between modules and patients’ clinical characteristics, we conducted Pearson correlation analysis.

### Construct predictive models through various machine learning methods

To obtain hub DEOSGs, we intersected the DEOSGs of the control group vs treat group and the C1 vs C2 clusters. Using these hub DEOSGs, we applied various R packages such as ‘caret’, ‘DALEX’, ‘randomForest’, ‘kernlab’ and ‘xgboost’ to establish four machine learning models - random forest (RF), support vector machine (SVM), generalized linear model (GLM) and extreme gradient boosting (XGB). To facilitate the selection of a machine learning model, we utilized box plots and ROC curves to display the outcomes of the four models.

### Construction a nomogram model

In order to facilitate the assessment of Alzheimer’s disease (AD) risk, this study utilized the ‘rms’ R software package to develop a nomogram model. The model is based on the hub genes of the Generalized Linear Model (GLM) machine learning model, which assigns scores to each gene. The total score is then used to determine the risk of disease occurrence. Additionally, the predictive ability of the model was evaluated using decision curve analysis (DCA) and calibration curves.

### Validation of predictive models

In this study, two brain tissue datasets, GSE122063 and GSE106241, were utilized to evaluate the effectiveness of predictive models in distinguishing Alzheimer’s disease (AD) from non-AD using the ‘pROC’ R package. Additionally, the relationship between hub DEOSGs and various immune cells was analyzed in GSE106241. To further understand the differential expression of these genes in AD and non-AD, their expression was visualized in both the training set and GSE106241. In GSE106241, the relationship between hub DEOSGs and age, Aβ-42 levels, and β-secretase activity was investigated using spearman correlation analysis. *P* < 0.05 were considered statistically significant.

### Experimental animals

A total of 30 8-month-old APP/PS1 mice and 10 same-line and same-month-old C57BL/6 wild-type mice, all SPF grade, were purchased from Beijing Huafukang Biotechnology Co., Ltd. The animals are kept in the Experimental Animal Center of Jinan University, with suitable temperature and humidity. All the animal experiments were reviewed and approved by the Experimental Animal Welfare Ethics Committee of Jinan University, Guangzhou.

### RNA collection, reverse transcription, and RT-qPCR

RNA was collected from temporal cortex of AD mice by the use of TRIzol (Invitrogen, USA). The RNA concentration was measured under Nanodrop ND-1000 spectrophotometry (Nanodrop Tech, USA) and RNA integrity was detected with denatured agarose gel electrophoresis. cDNA was acquired by reverse transcription using the SuperScript VILO cDNA Kit. The primers were constructed and synthesized by Sangon Biotechnology (Shanghai, China). RT-qPCR was conducted with the iQ5 RT-qPCR Detection System (Bio-Rad Laboratories, USA) following manufacturer’s instructions. In this, all the primers were listed in Table 1.

**Table 1:** Primer list.

**Gene**	**Primers**
NFKBIA	Forward: 5'-TCCACTCCATCCTGAAGGCTAC -3'
	Reverse: 5'- CAAGGACACCAAAAGCTCCACG -3'
PLCE1	Forward: 5'- AGAGCCTACTCTTTGACCACGG -3'
	Reverse: 5'- GTCTGAGACCATGAAGCTCTGG -3'
CLIC1	Forward: 5'- CCAGAGACTGTTCATGGTGCTC -3'
	Reverse: 5'- TCGGTGCCATAGAGCAAGAACG -3'
SLCO4A1	Forward: 5'- TGGCAAGACTGTCAGAGACCTG -3'
	Reverse: 5'- CGGCAATGAGTGTGGCTTCAGT -3'
TRAF3IP2	Forward: 5'- GTCATCCTGAATGACTCCAGCC -3'
	Reverse: 5'- GGAAGGTCCAAGGACTCCTCAG -3'
GAPDH	Forward: 5'- ATCACTGCCACCCAGAAGAC -3'
	Reverse: 5'- ACACATTGGGGGTAGGAACA -3'

### Immunohistochemistry (IHC)

The tissues were preserved with 4% paraformaldehyde (15 min), immersed in paraffin, and cut into an average of 4 μm slices. The antigens were extracted after dewaxing and dehydration. The completed slices were then fixed with 3% hydrogen peroxide for 20 min and blocked at room temperature for 15 min with 5% BSA. Whereafter, anti-NFKBIA (#4814; 1:100; CST), anti-PLCE1 (ab121476; 1:80; Abcam), anti-CLIC1 (ab219265; 1:100; Abcam), anti-SLCO4A1 (ab122124; 1:50; Abcam) and anti-TRAF3IP2 (ab5973; 1:100; Abcam) were incubated at 4° C overnight, using Antibody Diluent Solution (Life-iLab, Shanghai, China). The segments were colored by the color-developing agent for 3-15 min and then were washed, redyed, dehydrated, transparent, and sealed in sequence, which were detected by SP kits (Solarbio, Beijing, China). Finally, these slices were put under a light microscope for observation and photography.

### Measurement of ROS, MDA, GSH and oxidative stress index (OSI)

Intracellular ROS level was detected by the ROS Assay Kits (Beyotime; S0033; Haimen, China) according to the recommended manuals. The MDA and GSH levels were conducted using MDA Assay Kit (Beyotime; S0131; Haimen, China) and GSH Assay Kit (Nanjing Jiancheng Bioengineering Institute; A006-2-1; Nanjing, China) according to the instructions. Moreover, oxidative stress was deeply measured by the oxidative stress index (OSI) which was the ratio of TOS (Randox Laboratories, County Antrim, UK): TAC (Randox Laboratories, County Antrim, UK) (Bennett Plasma oxidative stress in reproduction of two eusocial African mole-rat species, the naked mole-rat and the Damaraland mole-rat).

### Statistical analysis

One-way analysis of variance (ANOVA) and the paired samples t-test were utilized to evaluate differences between groups, while Pearson’s correlation test was used to analyze correlations. Statistical analyses were performed using SPSS 25.0 software and GraphPad Prism 8.0.1. All data are presented as mean ± SD, and all experiments were conducted independently and repeated three times. A significance level of *P* < 0.05 was considered statistically significant.

### Data availability statement

The datasets were downloaded from the GEO (https://www.ncbi.nlm.nih.gov/geo/) in this study.
